# Randomized controlled trial of robot-assisted gait training with dorsiflexion assistance on chronic stroke patients wearing ankle-foot-orthosis

**DOI:** 10.1186/s12984-018-0394-7

**Published:** 2018-06-19

**Authors:** Ling-Fung Yeung, Corinna Ockenfeld, Man-Kit Pang, Hon-Wah Wai, Oi-Yan Soo, Sheung-Wai Li, Kai-Yu Tong

**Affiliations:** 1 Department of Biomedical Engineering, The Chinese University of Hong Kong, ShaTin, Hong Kong; 2 Department of Biomedical Engineering, The Hong Kong Polytechnic University, Hung Hom, Hong Kong; 3 Industrial Centre, The Hong Kong Polytechnic University, Hung Hom, Hong Kong; 4 Department of Medicine & Therapeutics, The Chinese University of Hong Kong, Ma Liu Shui, Hong Kong; 5 Division of Rehabilitation, Department of Medicine, The University of Hong Kong, Pok Fu Lam, Hong Kong

**Keywords:** Stroke, Robotics, Exoskeletons, Ankle foot orthosis, Gait training

## Abstract

**Background:**

Robot-assisted ankle-foot-orthosis (AFO) can provide immediate powered ankle assistance in post-stroke gait training. Our research team has developed a novel lightweight portable robot-assisted AFO which is capable of detecting walking intentions using sensor feedback of wearer’s gait pattern. This study aims to investigate the therapeutic effects of robot-assisted gait training with ankle dorsiflexion assistance.

**Methods:**

This was a double-blinded randomized controlled trial. Nineteen chronic stroke patients with motor impairment at ankle participated in 20-session robot-assisted gait training for about five weeks, with 30-min over-ground walking and stair ambulation practices. Robot-assisted AFO either provided active powered ankle assistance during swing phase in Robotic Group (*n* = 9), or torque impedance at ankle joint as passive AFO in Sham Group (*n* = 10). Functional assessments were performed before and after the 20-session gait training with 3-month Follow-up. Primary outcome measure was gait independency assessed by Functional Ambulatory Category (FAC). Secondary outcome measures were clinical scores including Fugl-Meyer Assessment (FMA), Modified Ashworth Scale (MAS), Berg Balance Scale (BBS), Timed 10-Meter Walk Test (10MWT), Six-minute Walk Test (SMWT), supplemented by gait analysis. All outcome measures were performed in unassisted gait after patients had taken off the robot-assisted AFO. Repeated-measures analysis of covariance was conducted to test the group differences referenced to clinical scores before training.

**Results:**

After 20-session robot-assisted gait training with ankle dorsiflexion assistance, the active ankle assistance in Robotic Group induced changes in gait pattern with improved gait independency (all patients FAC ≥ 5 *post-*training and 3-month *follow-up*), motor recovery, walking speed, and greater confidence in affected side loading response (vertical ground reaction force + 1.49 N/kg, peak braking force + 0.24 N/kg) with heel strike instead of flat foot touch-down at initial contact (foot tilting + 1.91°). Sham Group reported reduction in affected leg range of motion (ankle dorsiflexion − 2.36° and knee flexion − 8.48°) during swing.

**Conclusions:**

Robot-assisted gait training with ankle dorsiflexion assistance could improve gait independency and help stroke patients developing confidence in weight acceptance, but future development of robot-assisted AFO should consider more lightweight and custom-fit design.

**Trial registration:**

ClinicalTrials.gov NCT02471248. Registered 15 June 2015 retrospectively registered.

**Electronic supplementary material:**

The online version of this article (10.1186/s12984-018-0394-7) contains supplementary material, which is available to authorized users.

## Background

Stroke is caused by intracranial haemorrhage or thrombosis, which cuts off arterial supply to brain tissue and usually damages the motor pathway of the central nervous system affecting one side of the body. About half of the stroke survivors cannot walk at stroke onset, but they have 60% chance to regain independent walking after rehabilitation [1]. Reduced descending neural drive to the paretic ankle joint causes muscle weakness and spasticity, often accompanied with drop foot which is characterized by the foot pointing downward and dragging on the ground during walking [2, 3]. To maintain sufficient foot clearance in swing phase, people with dropped foot have to compensate either by hip hiking with exaggerated flexion in hip and knee joints, or circumduction gait with the body leaning on the unaffected side and the leg swinging outward through an arc away from the midline [4–6]. These inefficient asymmetric gait patterns hinder the walking ability and contribute to slower walking speed [7, 8], increasing risk of falling [9, 10], and greater energy expenditure [11]. Poor mobility results in sedentary lifestyle and limited physical exercise [12], which further deteriorates lower-limb functionality.

Foot drop can be managed using ankle-foot-orthosis (AFO), which is rigid or articulated ankle brace that controls ankle range of motion (ROM). Meta-analysis shows walking in conventional AFO has immediate or short-term beneficial effects on gait pattern and mobility of stroke patients, including an overall increase in ankle dorsiflexion throughout gait cycle, improvements in Functional Ambulatory Category (FAC), walking speed, and stairs-climbing speed [13–15]. Recent development in robot-assisted AFO demonstrates power assistance at ankle joint can facilitate walking of patients presenting with foot drop, by actively assisting ankle dorsiflexion for foot clearance in swing phase and minimizing occurrence of foot slap at initial contact [16–18]. Previous studies only evaluated the immediate effects of stroke patients walking in passive AFO [14, 15] or robot-assisted AFO [19, 20], but they were not sure whether any assistive effects could be carried over to unassisted gait after the patients had taken off the devices, i.e. the therapeutic effects.

Neuroscience studies suggest the brain is capable of altering its functions and structures for adapting to internal and external environment; an ability known as neuroplasticity [2, 21, 22]. Researches show intensive repetitive skill training can enhance neuroplasticity and promote motor relearning of stroke patients [23, 24], which is achievable utilizing robot-assistance in clinical setting. The Anklebot that was developed in MIT can provide power assistance to stroke patients performing repetitive voluntary ankle sagittal movements in seated position, and a single-arm pilot study reports stroke patients (*n* = 8) had improved volitional ankle control and spatial-temporal gait parameters after 6-week 18-session training using the Anklebot [25]; 30-min seated skill training at ankle joint can induce plastic changes in cortical excitability in area controlling dorsiflexor [26]. Thus robot-assisted AFO with dorsiflexion assistance can potentially stimulate motor recovery of stroke patients with foot drop problem. Neuroscience studies further show the functional outcome of neuroplasticity is task-specific and dependent on the training nature [2, 21, 22, 27]. It implies that in order to improve independent walking ability, stroke patients are expected to practise real over-ground walking instead of seated training. Incorporation of stair ambulation into gait training could facilitate generalization towards activity of daily-living, which requires stroke patients to perform skilled ankle dorsiflexion and plantarflexion when they are negotiating steps. Another characteristics of neuroplasticity is the importance of salient experiences for motor relearning from error correction [2, 21, 22]. During gait training, powered ankle assistance from a robot-assisted AFO could serve as a source of salient proprioceptive feedback synchronized to gait pattern [28]. The robot can strengthen the experience-driven neuroplasticity by producing this proprioceptive feedback at each successfully triggered ankle power assistance [28]. In summary, researches on experience-driven neuroplasticity suggest stroke patients presenting with foot drop problem can potentially restore some level of independent walking ability through robot-assisted gait training with ankle dorsiflexion assistance on over-ground walking and stair ambulation.

To our knowledge, up to now no randomized controlled trial (RCT) has been carried out to validate the rehabilitation approach of robot-assisted AFO [29, 30]. The current study aims to evaluate whether gait training with robot-assisted AFO with dorsiflexion assistance can bring greater improvement in independent walking ability than training with passive AFO. In each session, stroke patients were trained in 20-min over-ground walking and 10-min stair ambulation. Assessments on the participating stroke patients focused on functional changes in unassisted gait after they had discontinued to wear the devices, i.e. the therapeutic effects. A meta-analysis study recommends FAC to be the primary outcome measure for clinical trials involving electromechanical gait training [30]. FAC is a reliable measurement of independent walking ability on level ground walking and stair ambulation, which is a good prediction of independent community walking post-stroke [31]. The demonstration of safety and effectiveness of the robot-assisted gait training can have positive impact on post-stroke rehabilitation and can potentially establish a new treatment method for stroke patients presenting with foot drop.

## Methods

### Subjects

This was a double-blinded, parallel-group, randomized, sham-controlled clinical trial with 3-month follow-up conducted in Hong Kong between 2015 and 2017. Chronic stroke patients were screened for eligibility from local stroke community. Recruited subjects had mild motor impairment in affected ankle with drop foot gait abnormality. The presence of foot drop was assessed by asking the stroke patients to perform ankle dorsiflexion and observing if the ankle joint was unable to dorsiflex exceeding the neutral position. In addition, the stroke patients must be capable of standing and walking without manual assistance for extended period of time assessed by FAC ≥ 4 and Berg Balance Scale, BBS ≥ 40. Exclusion criteria were severe contractures that would preclude passive ROM in lower limb assessed by Modified Ashworth Scale, MAS ≥ 3. Stroke patients who were scheduled in other lower-limb therapeutic treatments during the prescribed training and *follow-up* period were rejected. After recruited subjects presented written informed consent, they were randomly assigned into Robotic Group or Sham Group in 1:1 ratio by the trainer drawing lots. All subjects and assessors kept blinded to the group allocation throughout the trial. The trainer delivering gait training did not involve in subject assessment.

### Intervention

Subjects stayed in the trial for about six months, including gait training and *follow-up* period. They received 20-session gait training for at least twice per week. The trainings were conducted in three centers with similar settings: having long (> 10 m) corridor cleared of obstacles with minimal turning points and staircase with handrail (≤10 steps, about 15 cm step height). The assigned training location alternated between sessions to allow subjects exposure to various surroundings for enhancing task variation. All trainings were delivered by the same skilled trainer despite multiple centers. In each session, subjects performed three 10-min walking tasks: (1) the first over-ground walking, (2) the staircase ascending/descending, and (3) the second over-ground walking. Subjects were free to take break and were allowed to use walking cane if necessary. Skilled trainer walked alongside the subject during gait training to ensure safety and to administer verbal cue on head/trunk extension in case of increasing trunk kyphosis, or mid-line awareness when the subject leaned on the unaffected side. The whole training session lasted within an hour including setup time.

### Robot-assisted AFO with dorsiflexion assistance

**Fig. 1:**
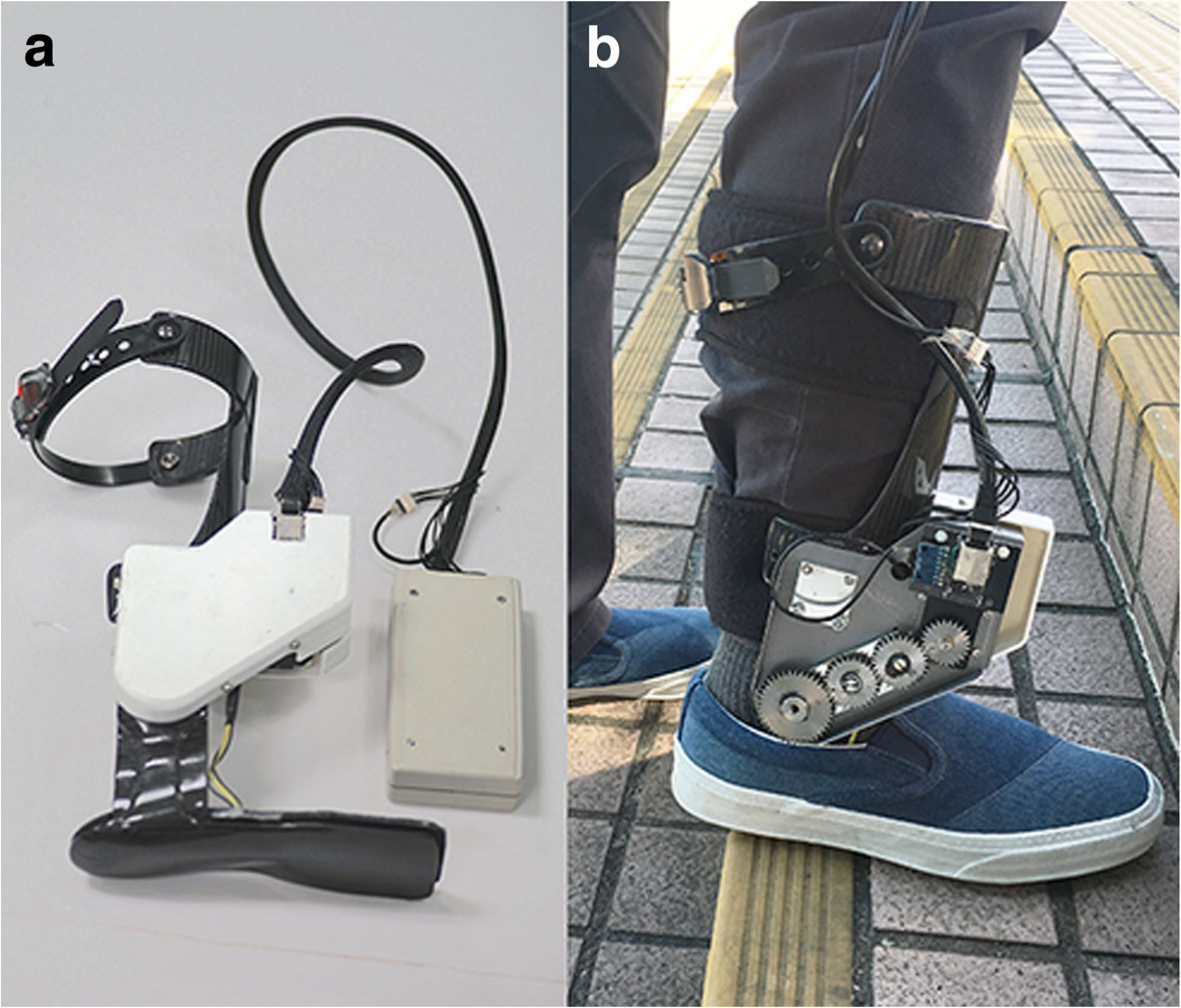
**a** Robot-assisted AFO, and **b** Stroke patients walking on stairs wearing the robot-assisted AFO

Detailed description of the robot-assisted AFO is presented elsewhere [32]. The robot-assisted AFO was developed by our research team, and was designed for long-distance walking to be portable without tethered external power source to facilitate casual walking in indoor environment with stairs (Fig. 1). The AFO was equipped with force sensitive resistors (FSR-402, Interlink Electronics, USA) under forefoot and heel to detect foot loading pattern. An inertial measurement unit (MPU6050 6-axis MotionTracking™, InvenSense, USA) with integrated accelerometer and gyroscope acquired shank movement data, which were inputs of a decision tree algorithm to classify three walking conditions: level-walk, stair-ascend, and stair-descend. Computation was performed using Arduino Pro Mini with ATmega328-5 V-16 MHz chip (Atmel, USA). Signals were sampled at 30 Hz, low-pass filtered at 4 Hz cut-off frequency. A brushless dc motor (Dynamixel MX106, ROBOTIS, South Korea) with built-in PID control actuated the ankle joint in one degree-of-freedom at maximum torque 16.7 Nm. A 12 V 1800mAh lithium polymer battery supplied power to both the electronic circuit and the motor. The battery capacity was sufficient for 5-h continuous training without recharging. Unlike other existing exoskeleton AFO, this robot-assisted AFO was designed to be wore inside wearer’s footwear like conventional AFO, and was fabricated using reinforced carbon fiber composite which is thin, lightweight but strong. Weight of the whole device was distributed to the waist (0.5 kg including battery and circuit board) and the ankle joint (0.5 kg including AFO and motor).

Robotic group received powered ankle assistance from the robot-assisted AFO with output torque in either dorsiflexion or plantarflexion direction depending on the classified walking conditions. An assisted-as-needed approach ensured the power assistance was just sufficient to assist the dropped foot together with subject’s voluntary residual joint movement to provide foot clearance (constant dorsiflexion assistance in swing phase) for over-ground walking and stair ascending, and controlled loading response (constant plantarflexion assistance from terminal swing to loading response) to prepare for foot landing on the lower step for weight acceptance in stair descending [33]. The torque output levels (ranged approximately 3.7 ± 2.1 Nm) were calibrated at the beginning of each session to adjust for any progression of functional changes throughout the 20-session gait training. Subjects stood quietly, relaxed and unloaded the dropped foot by shifting the body weight to the unaffected side. To calibrate dorsiflexion assistance, subjects were told to perform voluntary maximum ankle dorsiflexion on the dropped foot, while the motor torque was gradually increasing in dorsiflexion direction until the affected ankle joint angle reached 10° dorsiflexion. To calibrate plantarflexion assistance, subjects were told to stand quietly on both legs, then the motor torque increased gradually in plantarflexion direction until the torque was sufficient to uplift the heel on the affected side. In Sham Group, the robot-assisted AFO provided torque impedance to fix the ankle joint at neutral position, a condition similar to wearing rigid AFO. The robot-assisted AFO generated an audible electronic tone each time the power assistance was triggered, which was intended for the trainer to check whether the robot was working properly. Walking distance and stairs covered in each 10-min session were recorded as a reference of how much training dosage did the subjects receive.

### Outcome measures

Clinical assessments were carried out by blinded assessors at three time points to evaluate the therapeutic effects of gait training. (1) *Pre*: within a week before the first training session, (2) *Post*: within a week after the last training session, and (3) *Follow-up*: 3-month after the trainings ended. All clinical scores were assessed on patients without using any assistive devices, neither the robot-assisted AFO nor any orthosis the patients usually use. The same trained assessor administered the three assessments of a patient. The primary outcome measure was FAC, consisting of 6-level scale to assess functional independent walking on level or non-level surfaces: FAC = 4 requires supervision in level ground walking, FAC = 5 requires supervision only when walking on non-level surface such as stairs [31]. Secondary outcome measures were common clinical scores in evaluation of robot-assisted gait training [34], including: (1) Fugl-Meyer Assessment (FMA) for motor recovery, consisting of 34-score to examine lower-limb motor function quantitatively in reflex, flexor/extensor synergy pattern, volitional movement, coordination and speed; (2) MAS, consisting of 4-level scale to examine joint spasticity during passive muscle stretching; (3) BBS, consisting of 14-item objective measures to assess static balance for predicting falling risk; (4) Timed 10-Meter Walk Test (Timed 10MWT), to measure walking speed in short distance; (5) Six-Minute Walk Test (SMWT), to assess endurance using walking distance covered in fixed duration.

These six clinical assessment scores were supplemented by gait analysis to help explaining the changes in gait performance. Gait patterns when subjects walked over-ground without wearing any AFO were recorded at two time points: (1) *Pre* and (2) *Post*. A six-camera motion capture system (Bonita-10, Vicon, Oxford Metrics, USA) was used for three-dimensional motion analysis. Ground reaction forces (GRF) were measured using two force plates (Advanced Mechanical Technology, Inc., USA) embedded midway on a straight 6-m walkway. Walking trials were repeated until at least three samples of successful steps were collected, in which the whole foot completely landed within force plate under comfortable walking speed. All successful trials were used in the analysis. Kinematic and kinetic data were collected at 200 and 1000 Hz respectively; low-pass filtered using 4th-order Butterworth filter with 6 Hz cut-off frequency; and analyzed using inverse kinematics and inverse dynamics model of the BodyBuilder (Vicon, Oxford Metric, USA). Kinetic data were normalized to body mass. Local maxima and points of interest were determined within gait cycle and averaged across repeated trials. Foot tilting angle measures the absolute angle between the foot and the ground, which is negative when the foot is pointing downwards. This angle measurement can help identifying abnormality in foot orientation during walking, such as foot slapping at initial contact or dropped foot pointing downwards after mid-swing.

### Statistical analysis

The current study is intended to be a pilot trial of the robot-assisted AFO with dorsiflexion assistance being used in gait training, previous studies have few evidences of the effects on unassisted walking after subjects had discontinued wearing AFO [29, 30]. Expected benefit and risk of the recently developed robot-assisted AFO on chronic stroke patients are not available for sample size estimation. Taking into consideration ethical concerns and available resources [35], we referenced the sample size based on the single-arm pilot study of the Anklebot (*n* = 8) [25], which involved similar functional training at ankle except the training was carried out in seated position.

All outcome measures were analyzed based on the intention-to-treat principle, which included all randomized subjects. The last-observation-carried-forward method was used to impute the last available data to missing entries for subjects who did not complete the entire trial, i.e. dropout, and to avoid overestimation of clinical effectiveness. Analysis of covariance (ANCOVA) supplemented by repeated measure analysis was used to test the group difference and to reduce the probability of Type-I error owing to multiple comparisons. This is a technique for assessing group differences across metric-dependent variables based on categorical independent variables. Within-subject factor was set as time and the between-subject factor was set as group, with the ANCOVA model adjusted using the *Pre*-assessment parameters as covariate. If ANCOVA revealed significant effects, post-hoc comparison between assessment time points were tested using non-parametric Wilcoxon Signed Ranks Test. 95% confidence interval (95%CI) of statistical differences were calculated. We aim to evaluate whether the results demonstrate superiority of active assistance with robot-assisted AFO to Sham intervention with passive AFO. Two-tailed level of significance α set at 5%. All statistical analyses were computed by IBM SPSS Statistics for Macintosh, Version 23.0. (IBM Corp., USA).

## Results

**Fig. 2:**
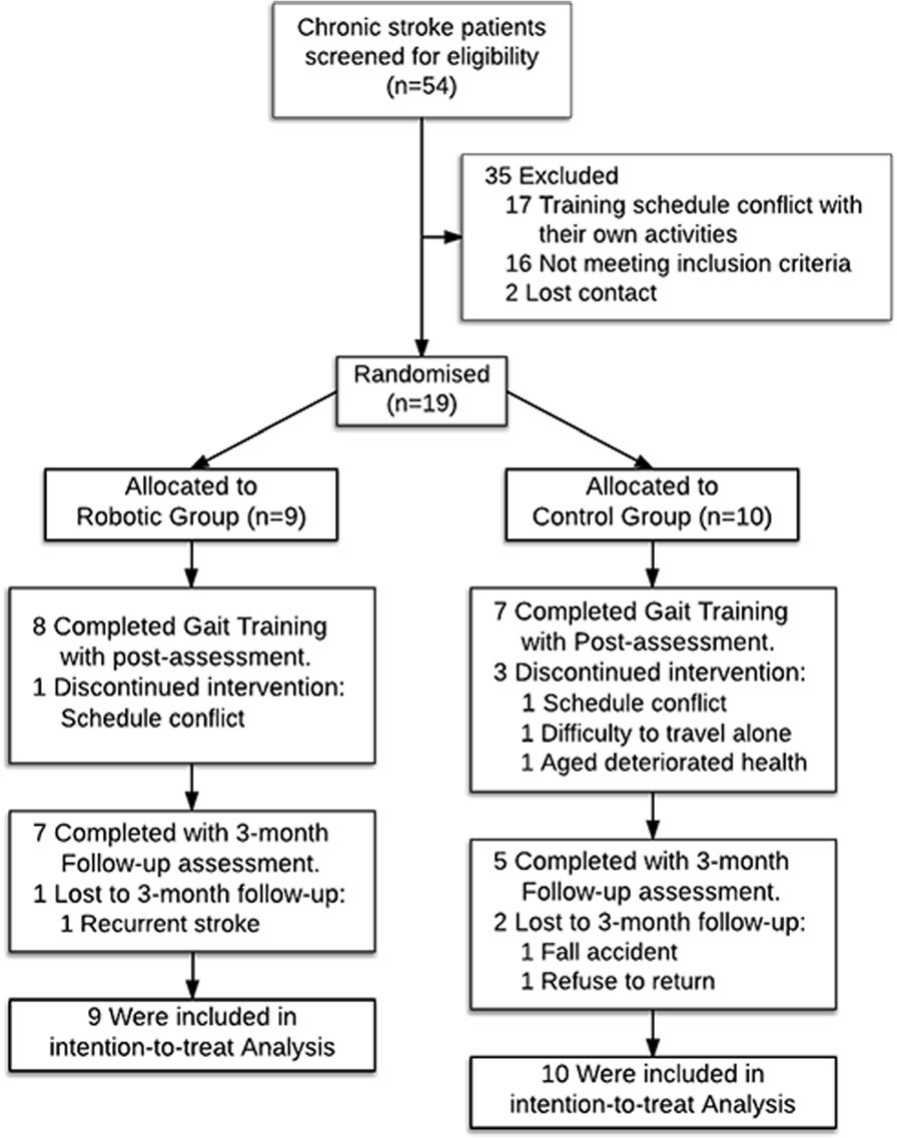
CONSORT patient flow diagram

Total 54 chronic stroke patients were screened for eligibility from June 2015 to November 2016. Nineteen met eligibility criteria were randomized and allocated into Robotic Group (*n* = 9) and Sham Group (*n* = 10). No statistically significant between-group difference was found in the demographic characteristics (Table 1) and the baseline values of the clinical scores (Table 2). Total four subjects discontinued intervention after they had participated in at least three training sessions; and three subjects lost to 3-month *follow-up*. Patient flow is presented in Fig. 2. No serious adverse event or important harm was reported.

**Table 1:** Demographic characteristics

Characteristics	All Participants (*n* = 19)	Robotic Group (*n* = 9)	Control Group (*n* = 10)	*p*
Age (years)^†^	57.9 ± 12.0	54.2 ± 13.0	61.2 ± 10.6	.931
Gender (male/female)	13/6	6/3	7/3	1.000
Affected side (left/right)	10/9	5/4	5/5	1.000
Stroke type (ischemic/hemorrhagic)	14/5	5/4	9/1	.141
Duration of stroke (years)^†^	5.2 ± 3.7	4.4 ± 2.5	6.0 ± 4.5	.103
Duration of gait training (weeks)^†^	5.3 ± 3.3	6.0 ± 2.6	4.7 ± 3.8	.259

^†^values present in mean ± SD

**Table 2:** Clinical assessment scores of lower-limb functionality at baseline (*Pre*) and the changes after the gait training (*Post*) with 3-month follow-up (*follow-up*). The significant changes between assessment time points, and the significant differences between groups (ANCOVA-adjusted to baseline *Pre*-assessment) are marked with asterisk

Outcome Measures^†^	Sham Group	Robotic Group	*Pre*-Adjusted Group Difference
	(*n* = 10)	(*n* = 9)	
FAC *(max. 6)*			
*Pre*	4.5 (0.8)	4.8 (0.8)	
*Post – Pre*	+ 0.1	+ 0.6 *	+ 0.50 *
*Follow-up – Pre*	+ 0.3	+ 0.7 **	+ 0.43
FMA *(max. 34)*			
*Pre*	16.1 (5.2)	17.9 (4.6)	
*Post – Pre*	+ 1.0	+ 2.4 *	+ 1.9 *
*Follow-up – Pre*	+ 1.3	+ 2.4	+ 1.9
MAS *(max. 4)*			
*Pre*	1.7 (0.6)	1.4 (0.5)	
*Post – Pre*	−0.1	− 0.2	− 0.22
*Follow-up – Pre*	− 0.2 *	− 0.2	− 0.08
BBS *(max. 56)*			
*Pre*	47.6 (5.4)	51.4 (5.1)	
*Post – Pre*	−0.2	+ 0.3	+ 0.04
*Follow-up – Pre*	−0.4	−0.8	− 0.28
Timed 10mWT *(m/s)*			
*Pre*	0.52 (0.37)	0.72 (0.21)	
*Post – Pre*	+ 0.01	+ 0.07 *	+ 0.05 *
*Follow-up – Pre*	+ 0.00	+ 0.10	+ 0.09
SMWT *(m)*			
*Pre*	141.6 (91.2)	207.3 (59.7)	
*Post – Pre*	+ 5.7	+ 16.9	+ 11.9
*Follow-up – Pre*	+ 22.2	+ 41.5	+ 7.0

*NOTE*: *FAC* Functional Ambulation Categories, *FMA* Fugl-Meyer assessment for Lower-Extremity, *MAS* Modified Ashworth Scale, *BBS* Berg Balance Scale, *10MWT* 10-Meter Walk Test, *SMWT* Six-Minute Walk Test

* *p* < 0.05; ** *p* < 0.01

^†^*Pre*-assessment values presented in mean (SD)

**Fig. 3:**
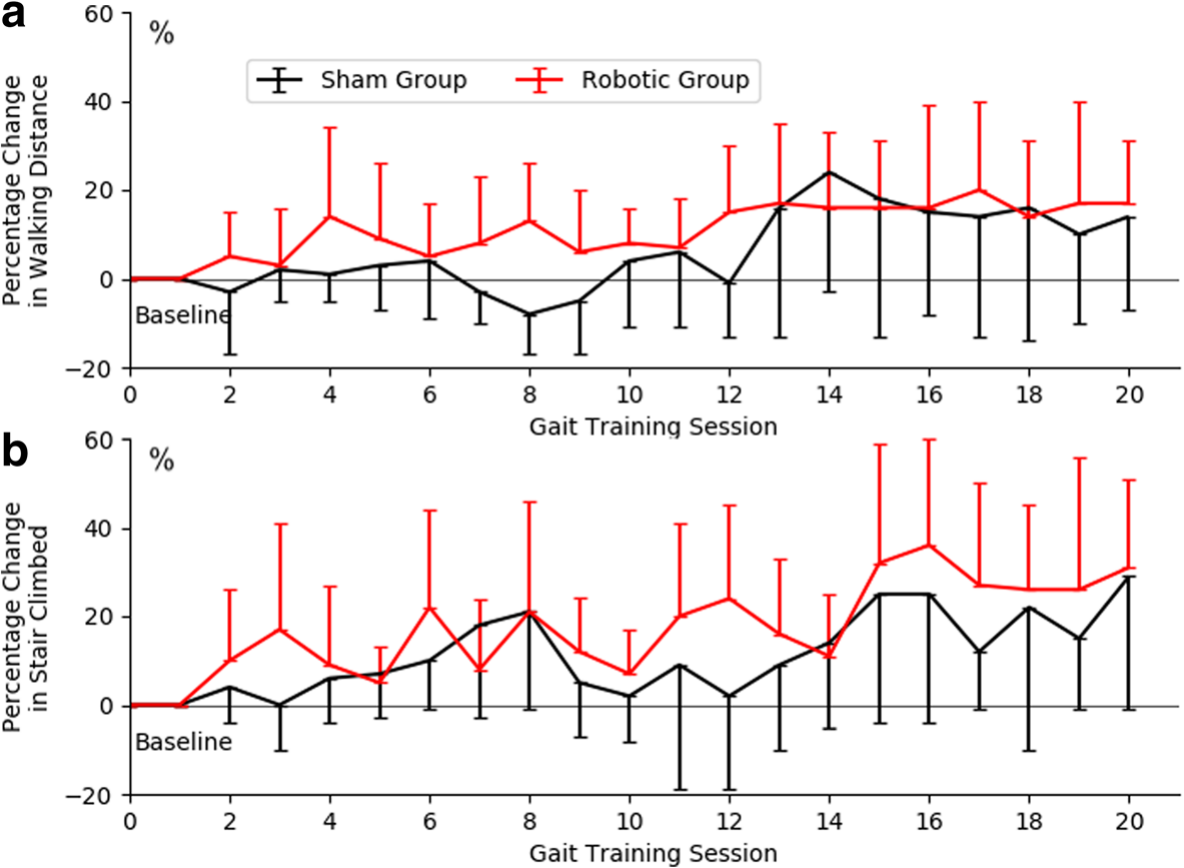
Percentage changes (mean ± SD) in (**a**) walking distance and (**b**) stairs covered across gait training sessions, normalized to the first session (Baseline)

The baseline 10-min over-ground walking distance were recorded as 287 ± 90 m in Robotic Group and 226 ± 145 m in Sham Group; the baseline 10-min stairs climbed were 257 ± 94 steps in Robotic Group and 204 ± 140 steps in Sham Group. Comparing the last training session (20th-session) with the baseline (1st-session), the walking distance recorded increased 17% in Robotic Group and 14% in Sham Group, the number of stairs covered increased 31% in Robotic Group and 29% in Sham Group (Fig. 3).

After the gait training, Robotic Group showed within-group improvement in FAC (+ 0.6, 95%CI: + 0.0/+ 1.1) which maintained in 3-month *follow-up* (+ 0.7, 95%CI: + 0.2/+ 1.2) (Table 2). Significant between-group difference in FAC *post-*training was + 0.5 (95%CI: + 0.1/+ 0.9) in favor to Robotic Group. More than half of the patients in Robotic Group showed improvements in gait independency and all reached FAC ≥ 5 *post-*training, while only one out of ten patients in Sham Group showed improved FAC *post*-training. On the other hand, the within-group increase in FMA (+ 2.4, 95%CI: + 0.4/+ 4.5) and the faster walking speed measured by Timed 10mWT (+ 0.07 m/s, 95%CI: + 0.0/+ 0.11) of Robotic Group *post*-training was significantly greater than that in Sham Group (between-group difference: FMA + 1.9, 95%CI: + 0.0/+ 3.9; walking speed + 0.05, 95%CI: + 0.0/+ 0.1); but the within-group changes were not statistically significant in the 3-month *follow-up* (FMA + 2.4, 95%CI: -2.2/+ 7.1; walking speed + 0.10 m/s, 95%CI: -0.04/+ 0.24). No significant between-group and within-group changes were found in MAS, BBS, and SMWT in unassisted walking.

**Fig. 4:**
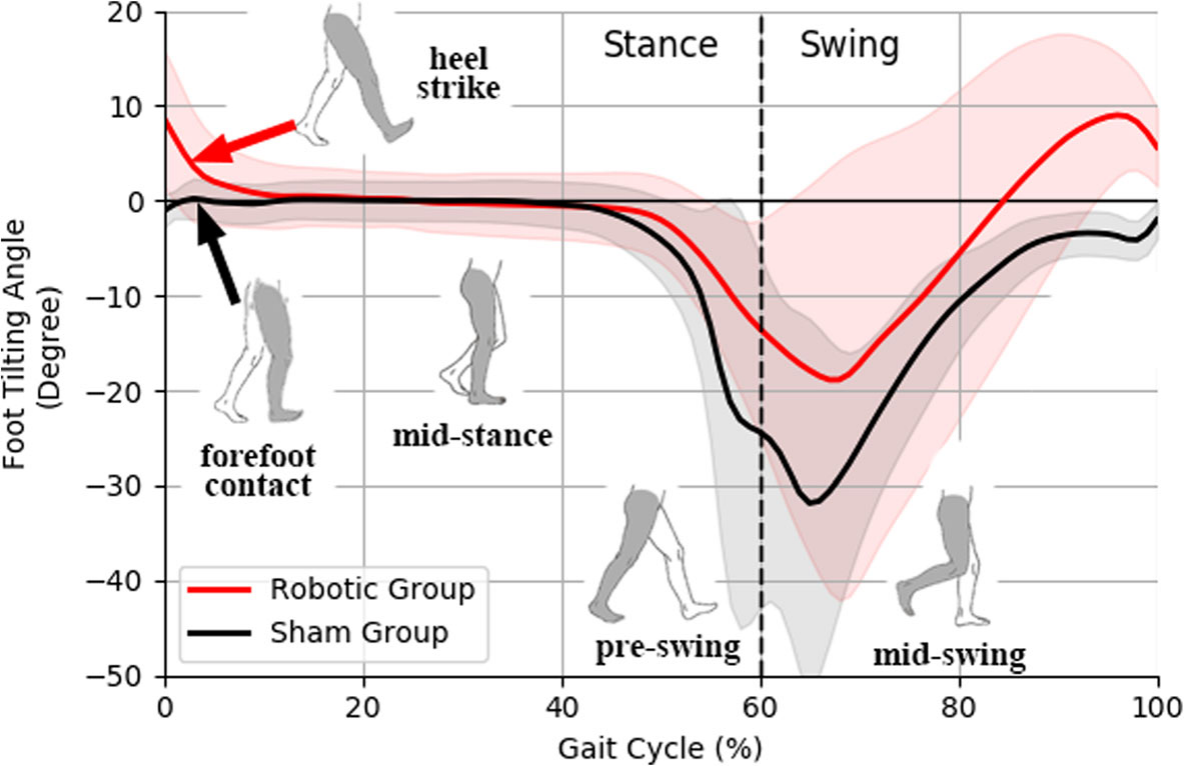
Foot tilting angle from the ground after gait training. Robotic Group (red) had positive tilting angle at initial contact for heel strike, while Sham Group (dark) used flat foot contact during weight acceptance (0–20% gait cycle). Foot tilting angle of Sham Group remained negative after mid-swing indicates foot drop abnormality

Some within-group differences were found in gait patterns after the gait training. Robotic Group walked faster (+ 0.07 m/s, 95%CI: + 0.0/+ 0.12). Analyses of spatial-temporal gait parameters show Robotic Group had shorter stance time on both affected side (− 0.24 s, 95%CI: -0.45/− 0.04) and unaffected side (− 0.40s, 95%CI: -0.73/− 0.08) after 20-session gait training (Table 3). Robotic Group showed significantly larger first peak of vertical GRF (+ 1.49 N/kg, 95%CI: + 0.71/+ 2.26) and peak braking force (+ 0.24 N/kg, 95%CI: + 0.02/+ 0.45) on affected side. At initial contact, the affected foot had positive tilting angle from the ground (heel strike the ground with foot pointing upwards) (+ 1.91°, 95%CI: + 0.42/+ 3.40) (Fig. 4). Affected side hip extension increased throughout gait cycle (+ 9.10° at stance, 95%CI: + 1.4/+ 16.8; and + 9.45° at swing, 95%CI: + 0.3/+ 18.6). Greater unaffected peak knee flexion (+ 9.5°, 95%CI: + 6.6/+ 12.4) was found during swing. After the 20-session gait training, Sham Group had longer stance time (+ 0.47 s, *Z* = 1.992) on affected side. Reduction in peak ankle dorsiflexion (− 2.36°, 95%CI: -3.95/− 0.77) and peak knee flexion (− 8.48°, 95%CI: -13.0/− 3.9) were found during swing in Sham Group. Foot tilting angle of Sham Group remained negative (dropped foot pointing downwards) after mid-swing (Fig. 4). Overall, Robotic Group placed significantly greater loading on affected side; and Sham Group had reduced affected leg ROM. Additional figures show the gait pattern in more detail (see Additional file 1).

**Table 3:** Spatial-temporal, kinetic, and kinematic gait parameters in gait analysis. The significant changes before (*Pre*) and after (*Post*) gait training, and the significant differences between groups (*ANCOVA*-adjusted to baseline *Pre* assessment) are marked with asterisk

Gait Parameters	Sham Group (*n* = 10)		Robotic Group (*n* = 9)		Group Difference in
	*Pre*	*Post-Pre*	*Pre*	*Post-Pre*	*Post-Pre* Changes
Walking Speed (m/s)	0.58 (0.17)	−0.06	0.74 (0.55)	+ 0.07 *	+ 0.06
Spatial-Temporal Gait Parameters					
Step Length (m)					
*Affected Side*	0.35 (0.12)	−0.04	0.32 (0.09)	+ 0.02	+ 0.04
*Unaffected Side*	0.33 (0.14)	−0.06	0.27 (0.12)	+ 0.01	+ 0.04
Stance Time (s)					
*Affected Side*	0.70 (0.27)	+ 0.47 *	0.74 (0.47)	−0.24 *	− 0.76
*Unaffected Side*	0.91 (0.33)	+ 0.35	0.97 (0.64)	−0.40 *	− 0.43
Swing Time (s)					
*Affected Side*	0.48 (0.16)	+ 0.15	0.38 (0.10)	−0.02	−0.14
*Unaffected Side*	0.24 (0.06)	+ 0.08	0.27 (0.11)	−0.03	−0.05
Peak Kinetic Gait Parameters (N/kg)					
Vertical Force @ loading response					
*Affected Side*	11.11 (2.23)	−0.52	8.96 (2.15)	+ 1.49 *	+ 1.26 *
*Unaffected Side*	10.35 (1.86)	+ 0.25	10.25 (1.58)	+ 0.60	+ 0.36
Vertical Force @ terminal stance					
*Affected Side*	9.83 (1.56)	+ 0.29	9.81 (1.95)	+ 0.13	−0.17
*Unaffected Side*	10.90 (2.23)	−0.40 *	10.10 (1.66)	+ 0.02	+ 0.45
Braking Force @ loading response					
*Affected Side*	0.81 (0.35)	+ 0.12	0.64 (0.22)	+ 0.24 *	+ 0.14
*Unaffected Side*	0.91 (0.66)	+ 0.01	0.65 (0.58)	+ 0.27 *	+ 0.28
Propulsive Force @ terminal stance					
*Affected Side*	0.61 (0.43)	−0.02	0.50 (0.33)	+ 0.10	+ 0.09
*Unaffected Side*	1.31 (0.65)	−0.30	0.80 (0.18)	+ 0.18	+ 0.30
Peak Kinematic Gait Parameters (Degree)					
Foot Tilting @ initial contact					
*Affected Side*	−1.19 (3.00)	−0.11	2.51 (4.18)	+ 1.91 *	+ 1.01
*Unaffected Side*	7.19 (4.48)	−3.37	7.69 (5.44)	−2.90	+ 2.51
Ankle Dorsiflexion @ stance					
*Affected Side*	16.0 (4.2)	+ 0.79	12.3 (4.07)	+ 0.72	−1.52
*Unaffected Side*	17.3 (4.1)	−0.58	13.5 (3.54)	+ 1.08	−0.72
Ankle Dorsiflexion @ swing					
*Affected Side*	1.62 (3.24)	−2.36 *	4.48 (4.22)	−1.00	−0.31
*Unaffected Side*	1.38 (4.68)	+ 0.71	5.39 (3.61)	+ 0.65	+ 0.84
Knee Flexion @ stance					
*Affected Side*	12.2 (10.7)	−6.41	17.3 (11.7)	−4.96	+ 3.83
*Unaffected Side*	27.3 (4.1)	−2.94	27.3 (4.6)	+ 3.49	+ 6.41
Knee Flexion @ swing					
*Affected Side*	23.9 (4.8)	−8.48 *	31.4 (16.3)	−3.86	+ 6.17
*Unaffected Side*	67.9 (5.4)	−3.74	60.9 (10.9)	+ 9.50 *	+ 15.5 *
Hip Flexion @ stance					
*Affected Side*	24.3 (10.7)	+ 0.32	31.2 (12.2)	−9.10 *	− 6.71
*Unaffected Side*	26.0 (11.5)	−4.91	16.6 (18.2)	+ 0.96	+ 6.60
Hip Flexion @ swing					
*Affected Side*	44.3 (18.1)	+ 2.03	53.1 (13.4)	−9.45 *	− 8.30
*Unaffected Side*	50.1 (10.5)	−1.18	41.8 (20.8)	+ 7.30	+ 5.90

*p < 0.05

## Discussion

The current study is one of the first RCTs investigating therapeutic effects of robot-assisted gait training with ankle dorsiflexion assistance for stroke patients with foot drop [29, 30]. After 20-session robot-assisted gait training involving over-ground walking and stair ambulation practices, Robotic Group demonstrated significant improvement in functional independent walking ability, motor recovery, and walking speed, compared with Sham Group. Gait analyses suggest stroke patients presenting with foot drop problem walked with greater gait confidence in affected side heel strike even after they had discontinued wearing the robot-assisted AFO, which might be associated with the patients adapting to the repetitive powered ankle dorsiflexion assistance. At 3-month *Follow-up*, the average values of the improvement in the Robotic Group maintained in gait independency, motor recovery, and walking speed, but there was no significant between-group difference at 3-month *Follow-up*.

Unrestricted over-ground walking practices that facilitate stroke patients to perform repetitive, voluntary, bipedal locomotion could be favorable to their gait recovery because of enhanced training intensity and active participation [2, 21, 22, 36, 37]. It could explain why in the current trial, both Robotic and Sham groups exhibited some degree of improvements in walking capacity and endurance across the 20-session gait training, which also implied increasing training dosage and adaptation to the gait assistance. The 17% improved walking capacity is greater than the minimal clinically important difference (MCID) of SMWT for walking endurance, which is 11.5% proportional changes in walking distance for stroke patients [38]. Few similar clinical studies evaluated the effects of robot-assisted gait training at ankle joint [30], including the powered AFO with robotic tendon developed in Arizona State University [20] and the H2 bilateral exoskeleton with ankle actuation [39]. Both exoskeleton robots have been evaluated on three chronic stroke patients in gait training sessions for at least three consecutive weeks. Researchers found positive results in SMWT with longer walking distance and stair climbed when subjects were walking in the exoskeleton robots [20, 39]. Although these two clinical studies did not have controls and had smaller sample size, results of the current pilot trial agrees with the previous studies [32].

Clinical assessments after the 20-session gait training show the Robotic Group had both within-group and between-group improvements in gait independency, motor recovery and walking speed, comparing with the Sham Group. FAC ≥ 5 indicates the Robotic Group became independent community walkers with minimal need of supervision on level ground walking and stair ambulation, which could be associated with faster walking speed [40]. The change in walking speed 0.07 m/s is greater than the minimal detectable change MDC = 0.05 m/s [41]. Nevertheless, these functional changes in motor recovery (FMA + 2.4) and walking speed (+ 0.07 m/s) were smaller than MCID of FMA = 6 [42] and MCID of walking speed = 0.16 m/s [43]. Similar to robot-assisted AFO with dorsiflexion assistance, the application of peroneal nerve functional electrical stimulation (FES) is also an alternative intervention to treat drop foot post-stroke. RCTs of FES show no significant superiority or inferiority over conventional AFO on treating stroke patients [15, 44]. The current study show robot assistance could improve gait independency, motor recovery, and walking speed of chronic stroke patients. Noting that both robot and FES assistance could lead to immediate improvement of walking endurance and functional ambulation [13, 14, 32], indicating these devices are good candidates of walking aids.

The improvement in the functional gait independency of Robotic Group suggests the subjects might benefit from changes in gait pattern that could facilitate their walking, which warrants further investigation in gait analysis. After 20-session gait training, Robotic Group exhibited positive foot tilting angle with foot pointing upwards on the affected side at initial contact, indicating heel strike (Fig. 4). The heel touch-down could help ankle dorsiflexors to act eccentrically for shock absorption, and could play an important role in smoothen transition into stance phase [2, 4, 10]. In affected side loading response, the larger first peak of vertical GRF and the greater braking force are correlated to the slightly faster walking speed as observed in the Robotic Group [45]. The improvement in weight acceptance indicates patients might develop confidence in standing on affected side after the robot-assisted gait training. Robotic Group walked with significantly shorter stance time on both sides but no significant change in swing time, which could imply the subjects spent shorter duration on double-support phase, and is a sign of improved dynamic gait stability and more confidence in shifting body weight. In addition, there were some interesting compensatory gait patterns, including the larger affected hip extension throughout gait cycle could be associated with more upright posture with better stability, and the larger unaffected knee flexion during swing could maintain important forward momentum [2, 4, 10]. During robot-assisted gait training, the highly repetitive and salient sensory feedback, such as the proprioceptive stimulation from the assisted ankle dorsiflexion movements and the pressure sensation from the mechanoreceptors under foot soles, are potential stimulants to strengthen induced plastic changes in gait pattern [21, 22]. Neuroscience researches demonstrate motor training with voluntary drive and appropriate afferent feedback could stimulate changes in motor cortex excitability [30, 37]. In future study, neuroimaging using MRI could further enhance the understanding of neuroplasticity in gait relearning of stroke patients.

Surprisingly from our results, gait analyses of Sham Group after long-term gait training reports reduction in affected ankle dorsiflexion and reduced knee flexion after subjects had taken off the passive AFO. These responses are unexpected since they are against the main purpose of the passive AFO, which is to provide support in foot clearance [13, 14]. Although efforts have been made to minimize the weight of the unpowered AFO, which is relatively more lightweight comparing with robotic AFO design from other research groups, it should be noted that both intervention groups still had extra 0.5 kg loading on affected ankle with additional metabolic cost and mechanical constraints [46], which could be the cause of the reduction in affected side ROM and the negative foot tilting angle (dropped foot pointing downward) maintained after mid-swing (Fig. 4). 3.6 kg unilateral loading of the MIT Anklebot would reduce peak dorsiflexion angle by about 7° at terminal stance [47], and 4 kg load mass on foot segment would increase metabolic cost of walking by 48% [48]. Previous studies demonstrate adding 2.5 kg mass on leg for a short duration did not alter lower-limb kinematics [46], yet our results suggest prolonged wearing 0.5 kg weight at affected ankle would still alter gait pattern even after the patients had taken off the device, which is an important limitation of the robot-assisted AFO. Moreover, participants complained about occasional poor fitting and misalignment of the robot-assisted AFO that pressed on the leg, particularly for patients with extreme body sizes, apparently customized AFO should be considered in future studies. Other studies on powered AFO also highlighted the importance of custom-fit exoskeleton for better comfort, stability, and robustness [20]. Future design of robot-assisted AFO should take into considerations the potential drawback of added mass, external perturbations, and custom-fit orthosis on ankle joint.

There are limitations in the current study. Firstly, we did not restrict the use of walking cane in the gait training due to safety concern. Most of the subjects carried the walking cane as a habit to feel more secure during walking, but over-reliance on the assistive device could affect motor relearning. Secondly, although the improvements in motor recovery and walking speed of the Robotic Group maintained in 3-month *follow-up*, the within-group differences were not statistically significant due to larger inter-subject variability in 3-month *follow-up* reduced the effect sizes. Since the intention-to-treat principle with the last-observation-carried-forward method was used in statistical analysis, the dropout cases in 3-month *follow-up* might affect the treatment effects. Larger sample size is required in future RCT studies of robot-assisted gait training. Thirdly, this RCT focused on chronic stroke patients with relatively mild impairment, so the clinical assessments were potentially limited to ceiling effects. Clinical study suggests the rate of motor recovery is higher in sub-acute phase than in chronic phase [1]. It is suggested sub-acute patients should perform more gait training in order to prepare for better motor outcome at discharge from hospital, especially when time spent in hospital therapy is limited [30]. Future studies should target at larger RCT of the robot-assisted gait training in sub-acute stroke.

## Conclusion

This RCT demonstrates that the robot-assisted gait training with ankle dorsiflexion assistance could improve functional gait independency, motor recovery, and walking speed of chronic stroke patients presenting with foot drop. Gait analyses show the participated stroke patients walked with greater confidence in weight acceptance using affected side heel strike. The intensive repetitive active ankle assistance might have enhanced motor relearning through neuroplasticity. The robot-assisted AFO is recommended as an assistive device for walking, but further development should be optimized towards more lightweight and custom-fit designs.

## Additional file

Additional file 1: Figures of the kinetic and kinematic gait pattern *pre*-*post* gait training. Figures showing the ground reaction forces (in fore-aft and vertical directions) and joint angles (in hips, knees, and ankles) of both intervention groups before and after the 20-session gait training. (PDF 318 kb)

## Abbreviations

95%CI: 95% Confidence Interval
AFO: Ankle Foot Orthosis
ANCOVA: Analysis of Covariance
BBS: Berg Balance Scale
FAC: Functional Ambulatory Category
FES: Functional Electrical Stimulation
FMA: Fugl-Meyer Assessment
GRF: Ground Reaction Force
MAS: Modified Ashworth Scale
MCID: Minimal Clinically Important Difference
MDC: Minimal Detectable Change
RCT: Randomized Controlled Trial
ROM: Range of Motion
SMWT: Six-Minute Walk Test
Timed 10MWT: Timed 10-Meter Walk Test
