# Supplementary material for: Randomized controlled trial of robot-assisted gait training with dorsiflexion assistance on chronic stroke patients wearing ankle-foot-orthosis
Source: J Neuroeng Rehabil. 2018 Jun 19;15:51. doi: 10.1186/s12984-018-0394-7 (PMC6006663; doi:10.1186/s12984-018-0394-7)
Supplement: Supplementary file 1 — Figures of the kinetic and kinematic gait pattern pre-post gait training. Figures showing the ground reaction forces (in fore-aft and vertical directions) and joint angles (in hips, knees, and ankles) of both intervention groups before and after the 20-session gait training. (PDF 318 kb) [file 12984_2018_394_MOESM1_ESM.pdf]

Supplemental Figures

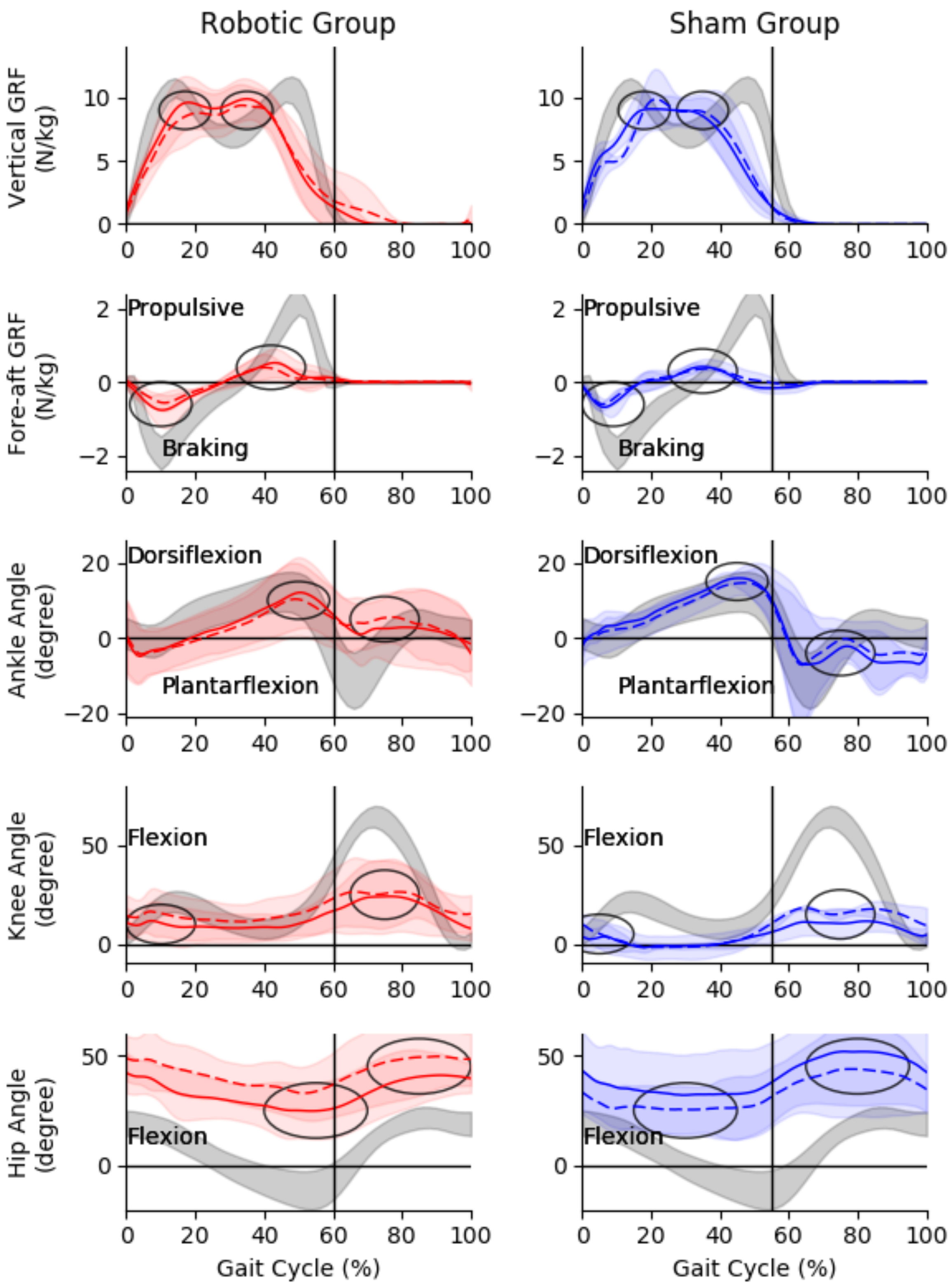

FIGURE I. Affected side kinetic and kinematic gait pattern in the gait analysis before (Pre: dashed line) and after (Post: solid line) the gait training. Local maxima and point of interests were circled. Gait patterns of healthy subject were shaded in grey for reference.

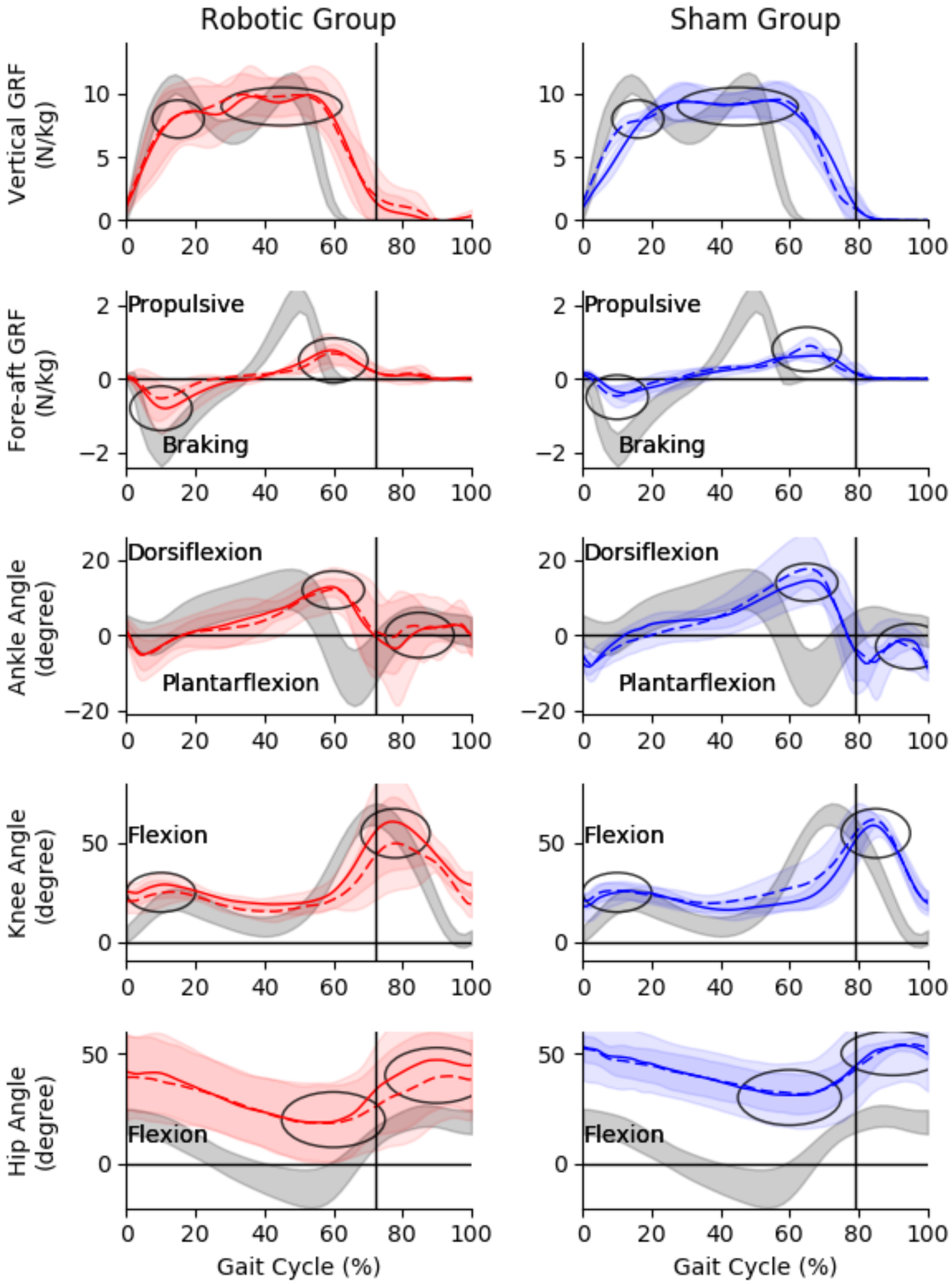

FIGURE II. Unaffected side kinetic and kinematic gait pattern in the gait analysis before (Pre: dashed line) and after (Post: solid line) the gait training. Local maxima and point of interests were circled. Gait patterns of healthy subject were shaded in grey for reference.
